# Effectiveness and safety of endoscopic submucosal dissection for residual or recurrent colorectal neoplasia: Meta-analysis

**DOI:** 10.1055/a-2606-0982

**Published:** 2025-07-01

**Authors:** Maximilian Eisele, Alessandra Ceccacci, Mehul Gupta, Emily Heer, Sherif Elhanafi, Saowanee Ngamruengphong, Nirav Thosani, Jordan Iannuzzi, Puja Kumar, Paul Belletrutti, Richdeep Gill, Nauzer Forbes

**Affiliations:** 1 2129 Medicine, University of Calgary, Calgary, Canada; 2 7938 Medicine, University of Toronto, Toronto, Canada; 3 2129 Medicine, Division of Gastroenterology, University of Calgary, Calgary, Canada; 4 6177 Division of Gastroenterology, Texas Tech University, Lubbock, United States; 5 1466 Gastroenterology and Hepatology, Johns Hopkins University, Baltimore, United States; 6 12339 Gastroenterology, Hepatology and Nutrition, University of Texas McGovern Medical School, Houston, United States; 7 2129 Surgery, University of Calgary, Calgary, Canada

**Keywords:** Endoscopy Lower GI Tract, Polyps / adenomas / ..., CRC screening, Endoscopic resection (polypectomy, ESD, EMRc, ...)

## Abstract

**Background and study aims:**

Endoscopic submucosal dissection (ESD) is a potentially surgery-sparing technique for colorectal neoplasia resection. Outcomes of ESD for residual or recurrent colorectal neoplasia are not well described. This meta-analysis aimed to evaluate the effectiveness and safety of ESD in treating residual or recurrent colorectal neoplasia.

**Patients and methods:**

We searched MEDLINE and Embase up to July 24, 2023 for studies on ESD for residual or recurrent colorectal neoplasia at prior surgery or endoscopic resection sites. The primary outcome of the meta-analysis was R0 resection; secondary outcomes included recurrence, adverse events (AEs), procedure time, and hospitalization length. Pooled effect sizes were obtained using inverse variance random effects models. Subgroup analyses were based on study location, lesion size, and endoscopist experience.

**Results:**

From 1,133 abstracts, data from 25 observational studies were included, reporting on 863 residual or recurrent lesions treated with ESD. R0 resection was achieved in 80.7% of patients (95% confidence interval 72.7–86.7%, I ^2^ = 81%) of patients, whereas recurrence occurred in 2.0% (0.7–5.1%, I ^2^ = 0%). Incidence of delayed bleeding and delayed perforation were 1.8% (0.7–4.2%, I ^2^ = 0%) and 1.9% (0.6–6.3%, I ^2^ = 35%), respectively. The former was independent of country of study, recurrent lesion size, or endoscopist experience. Mean procedure duration was 80.4 minutes (66.6–94.2, I ^2^ = 96%) and hospitalization length was 4.2 days (2.0–6.4, I ^2^ = 98%).

**Conclusions:**

This meta-analysis suggests that ESD is effective and safe for treating residual or recurrent colorectal neoplasia after previous resection, with further prospective validation studies needed to compare ESD with other endoscopic resection methods and surgery in this context.

## Introduction

Colorectal cancer (CRC) represents a significant global health burden 1 . Diagnoses of CRC are on the rise, including in younger patients 2 . However, advancements in screening, detection, and resection techniques, when applied appropriately, can facilitate early detection of premalignant colorectal neoplasms and lead to improved outcomes 3 4 . Endoscopic resection has emerged as a key strategy for management of early-stage colorectal lesions, offering a minimally invasive and safer alternative to surgery 5 . Techniques such as endoscopic mucosal resection (EMR) and endoscopic submucosal dissection (ESD) allow for removal of premalignant polyps and, in some cases, early-stage tumors, reducing the need for surgery 6 .

EMR and ESD are each associated with advantages and disadvantages 7 8 9 10 . Although experience with and guidance on ESD in the setting of premalignant and early malignant lesions of the upper gastrointestinal tract are widely available 11 12 13 , specific guidance on primary use of ESD in lower gastrointestinal lesions is relatively lacking, with a relative paucity of data informing conclusions regarding its safety in residual or recurrent colorectal lesions. Furthermore, with more and more colorectal lesions being treated endoscopically, and patients living longer in general, treatment of recurrences after primary resection is becoming a growing challenge. Residual or recurrent lesions can potentially pose further challenges to endoscopists due to fibrosis and scar formation, which can inhibit submucosal lifting and prevent successful endoscopic resection.

According to Japan Gastroenterological Endoscopy Society guidelines, treatment of residual and recurrent neoplasia in the colorectum should proceed en bloc 6 , which, in theory, leaves either ESD or surgical resection as the only options, with the former being less invasive and therefore preferred in terms of adverse events (AEs), but only if there is comparable effectiveness to surgery. Despite this, few studies have investigated effectiveness and safety of ESD in residual or recurrent colorectal lesions. Thus, we aimed, via a comprehensive systematic review and meta-analysis, to evaluate effectiveness and safety of ESD he treatment of residual or recurrent colorectal neoplasia.

## Methods

### Overview and objectives

This study was designed as a systematic review and meta-analysis. We followed the Preferred Reporting Items for Systematic Reviews and Meta-Analyses (PRISMA) statement ( **Supplementary Table 1** ) in this report and its protocol was initially registered on the International Prospective Register of Systematic Reviews (PROSPERO - CRD42023434171). The objectives of this study were to evaluate effectiveness and safety of ESD in treatment of residual or recurrent colorectal neoplasia. Given our study design and reliance upon previously published non-identifying data, neither institutional research board approval nor written consent were sought.

### Search strategy

We searched the electronic databases MEDLINE and Embase from inception to July 24, 2023 for suitable abstracts, and this search was then updated on January 3, 2024. We further manually searched reference lists of included studies, conference proceedings (Digestive Diseases Week, American College of Gastroenterology, and European Society of Gastrointestinal Endoscopy Days meetings between 2019 and 2023), and reviews for additional abstracts. Full details of our electronic search strategy are provided in **Supplementary Table 2** .

### Eligibility criteria and study selection

Our aim was to identify studies of adult patients having undergone ESD to resect residual or recurrent colorectal neoplasia, with at least one measure of effectiveness, safety, or recurrence reported. Both index and residual and recurrent lesions could be of any size and in any location in the colon or rectum. The index lesion could have been removed by any endoscopic or surgical technique. Therefore, studies were excluded if: 1) they did not report on resection residual or recurrent lesions; 2) they reported outcomes of only metachronous lesions, whose definitions are inconsistent and could occur at a different site than an index lesion; 3) they did not employ ESD as the endoscopic technique of choice for removal of the recurrent/residual lesion; 4) lesions were located in the upper gastrointestinal tract; 5) > 10% of lesions assessed were neuroendocrine tumors or scars (i.e.: non- adenomatous or early cancerous lesions); 6) participants had inflammatory bowel disease or 7) they were case reports.

Two authors (ME and AC) independently screened articles retrieved from the title/abstract search, with two votes of “include” resulting in the record moving forward with full-text abstraction, which was also performed in duplicate by the same two authors. Conflicts from either stage were resolved via consensus with other authors (NF and MG).

### Data extraction and quality assessment

Data were independently extracted by two authors (ME and AC) into data extraction forms ( **Supplementary Table 3** ), with conflicts being handled by consensus (between ME, AC, NF, and MG). Extracted data included: 1) basic study information, including year of publication, country/countries in which the research occurred, and study design; 2) participant characteristics, including age and gender; 3) initial lesion size, location, histology, and morphology; 4) recurrent lesion size, location, histology, morphology, and degree of fibrosis; 5) rates of complete, curative, and R0 resection; 6) AEs, including immediate and delayed bleeding, immediate and delayed perforation, infection, need for salvage surgery immediately following endoscopy, and need for additional surgery; 7) second recurrence rate; and 8) procedure and post-procedure details including procedure time, endoscopic techniques and tools, endoscopist training level, and length of subsequent hospitalization, if any. Risk of bias was assessed by two authors (ME and AC) via the Risk of Bias In Non-randomized Studies-of Interventions (ROBINS-I) tool for observational studies 14 .

### Outcomes and definitions

The primary outcome of our meta-analysis was R0 resection rate, defined as en bloc tumor resection with histological confirmation of both negative deep and lateral margins. Secondary outcomes of our study included: 1) en bloc resection rate, defined as tissue resected in one piece; 2) curative resection, defined as early cancer that was resected en bloc with negative horizontal and vertical margins and without lymphatic and vessel involvement while being limited to the submucosal layer; 3) local second recurrence rate, defined as early adenoma or cancer located at the site of prior resection or anastomosis; and 4) procedure time. Secondary outcomes related to safety included: 1) intra-procedure bleeding; 2) delayed bleeding (presenting greater than 24 hours post-procedure); 3) intra-procedure perforation; 4) delayed perforation; 5) need for salvage surgery (immediately following endoscopy); 6) need for additional surgery (at any point following the endoscopic procedure); 7) infection; and 8) length of hospitalization. The intent was to compare ESD with other modalities (EMR or surgery) in treating secondary lesions; however, due to a paucity of comparison trials, there was insufficient power to complete this analysis.

### Statistical analysis

Pooled measures of effect were obtained using a generalized linear mixed-effects model for dichotomous outcomes and reported as odds ratios with respective 95% confidence intervals (CIs). Heterogeneity was measured utilizing the Cochrane *I* ^2^ statistic. Publication bias was assessed by visual inspection of funnel plots and by Egger tests. To investigate sources of potential heterogeneity and control for potential confounders, several subgroup analyses were planned a priori, including: 1) Asian studies compared to non-Asian studies; 2) procedures performed by endoscopists with ≥ 100 independent ESDs performed compared with < 100; and 3) recurrent lesion size < 40 mm compared with ≥ 40 mm. All analyses were carried out in R version 4.3.2 (R Group for Statistical Computing, Vienna, Austria).

## Results

### Study selection and characteristics

**Fig. 1:**
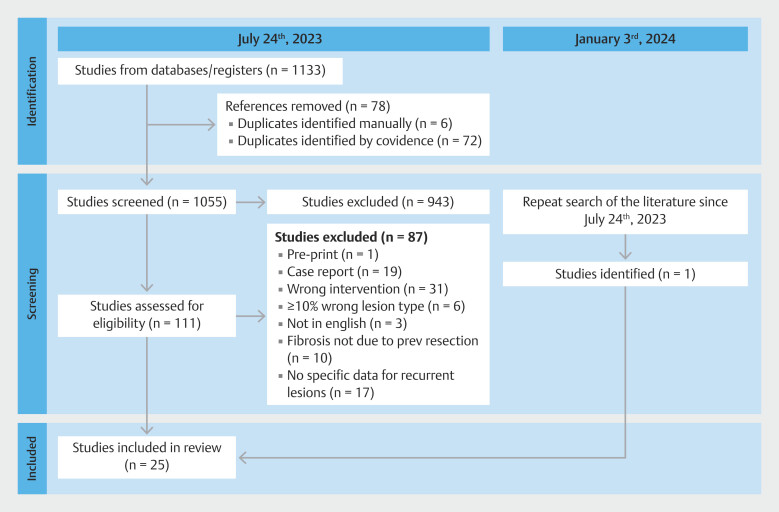
PRISMA flow diagram outlining the study identification, screening, and inclusion process 37 .

Our initial search yielded 1,133 records. Of these, 25 were ultimately included in the meta-analysis. The full study selection process is outlined in Fig. 1 . Fifteen studies were performed in Asia (Japan and China) 15 16 17 18 19 20 21 22 23 24 25 26 27 28 29 30 with the remainder performed in Europe 31 32 33 34 35 36 , the United States 34 , and Australia 34 . The earliest study began in 2003 and the most recent was completed in 2021, with all studies being published between 2008 and 2023. All studies were observational with 18 retrospective studies and seven prospective (including 2 with post-hoc analyses of prospectively maintained databases). Of the 25 studies, 19 had treatment of residual or recurrent lesions with ESD as their primary outcome. Overall, a total of 863 residual or recurrent colorectal lesions treated with ESD were included in the meta-analysis, with initial resection techniques including EMR, ESD, and surgery (including transanal endoscopic microsurgery). Mean or median lesion sizes ranged from 10.0 to 42.5 mm, and lesions were fairly evenly distributed across various locations in the colon and rectum. Median or median follow-up periods ranged between 6 and 66 months. A comprehensive overview of study and lesion characteristics from included studies is provided in Table 1 .

**Table:** **Table 1** Study, patient, and lesion characteristics among studies reporting on patients undergoing endoscopic submucosal dissection (ESD) for residual or recurrent colorectal neoplasia.

**First author**	**Year**	**Country(ies)**	**Number of lesions**	**Prior resection technique(s)**	**Male sex (%)**	**Mean/median age (years)**	**Mean/median neoplasia size (mm)**	**Primary lesion location(s)**	**Mean/median follow-up (months)**
Azzolini	2011	Italy	11	EMR	45.5	58	35.0	N/R	19
Cong	2016	China	11	ESD	N/R	N/R	N/R	N/R	N/R
Faller	2020	France	53	EMR	50.9	70	40.0	Right-sided	6
Gurram	2020	USA	3	EMR	66.7	63	42.5	Cecum	22
Hurlstone	2008	UK	30	EMR	72.0	65	26.0	Left-sided	12
Ikezawa	2021	Japan	10	TEM	33.3	76	27.5	Rectum	30
Ito	2019	Japan	26	ER	N/R	N/R	23.0	N/R	N/R
Krutsri	2019	Japan	4	Surgery	100.0	69	34.0	Rectum	12
Kuroki	2010	Japan	34	EMR	67.7	66	20.3	Mixed	12
Makazu	2015	Japan	2	ESD, EMR	57.1	66	N/R	N/R	N/R
Ohata	2022	Japan	3	ESD	N/R	N/R	> 10.0	N/R	N/R
Ohmori	2021	Japan	21	ER	75.0	72	15.0	Rectum	24
Pecere	2021	Japan	4	ER, surgery	73.3	67	30.0	Rectum	N/R
Rahmi	2015	Japan	28	ER	53.6	67	17.5	Mixed	22
Sakamoto	2011	Japan	9	ER	N/R	N/R	25.3	N/R	6
Spadaccini	2022	Europe/USA	81	ESD	69.1	70	41.0	Left-sided	30
Spychalski	2019	Poland	70	N/R	55.7	65	34.8	Left-sided	N/R
Suzuki	2019	Japan	27	EMR	N/R	72	20.0	Left-sided	33
Tanaka	2021	Japan	102	EMR, ESD	54.9	N/R	20.0	Mixed	33
Tanaka	2023	Japan	54	EMR, ESD	63.0	70	16.0	Mixed	60
Urban	2016	Czech Republic	5	EMR	40.0	69	N/R	N/R	6
Wang	2023	China	55	Surgery	61.8	63	17.0	N/R	66
Yang	2019	USA	27	EMR	N/R	N/R	N/R	N/R	N/R
Yzet	2023	France	177	ER, FTR	57.1	70	35.0	Mixed	N/R
Zhou	2009	China	16	EMR	56.3	65	N/R	Left-sided	16
ESD, endoscopic submucosal dissection; EMR, endoscopic mucosal resection; ER, endoscopic resection; FTRD, full-thickness resection; N/R, not reported; TEM, transanal endoscopic microsurgery.									

### R0 resection

**Fig. 2:**
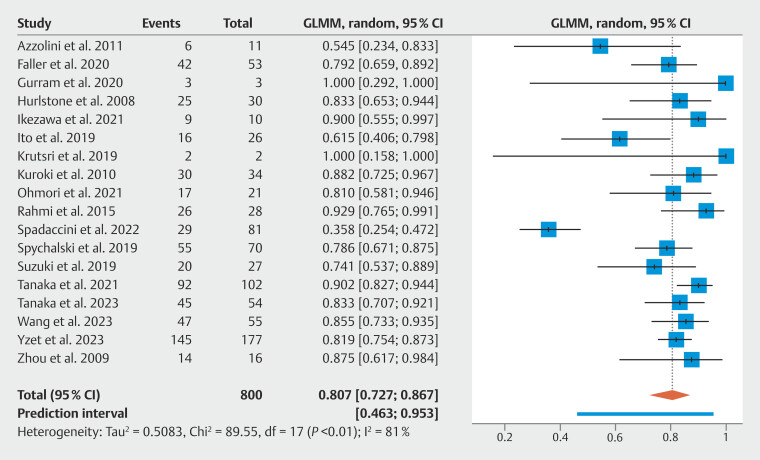
Forest plot of the proportion of successful R0 resections in patients undergoing endoscopic submucosal dissection for residual or recurrent colorectal neoplastic lesions.

A summary of pooled estimates for primary and secondary outcomes along with subgroup analyses is provided in Table 2 . Among 18 studies reporting on 800 residual or recurrent lesions treated with ESD, the pooled R0 resection rate was 80.7% (95% CI 72.7–86.7%). There was considerable heterogeneity between studies ( *I* ^2^ = 81.0%). The forest plot for the primary outcome is provided in Fig. 2 . There was no statistically significant difference in R0 resection rate ( *P* = 0.08) between studies conducted in Asia (84.6%, 95% CI 78.0–95.0%, *I* ^2^ = 38%) compared with those conducted outside of Asia (73.2%, 95% CI 53.7–86.5%, *I* ^2^ = 90%). Neither lesion size (≥ 40 mm vs < 40 mm) nor cumulative ESD experience of the performing endoscopist (≥ 100 vs < 100 ESD procedures) were statistically significantly correlated with R0 resection ( Table 2 ).

**Table:** **Table 2** Pooled estimates of primary and secondary outcomes with subgroup analyses based on study location, endoscopist experience, and lesion size.

**Outcome**	**Number of included lesions**	**Pooled estimate (95% CI)**	** Measures of hetero-geneity (I², χ² *P* value) **	**Study location**			**Endoscopist experience level**			**Lesion size**		
				**Non-Asia**	**Asia**	** Subgroup differences( *P* value) **	**< 100 ESD procedures**	**≥ 100 ESD procedures**	** Subgroup differences ( *P* value) **	**< 40 mm**	**≥ 40 mm**	** Subgroup differences ( *P* value) **
R0 resection rate (%)	800	80.7 (72.7–86.7)	81%, < 0.01	73.2 (53.7–86.5)	84.6 (78.0–95.0)	0.08	80.6 (18.8–98.7)	84.0 (76.2–89.4)	0.44	82.5 (77.6–86.6)	68.1 (7.0–98.4)	0.31
En bloc resection rate (%)	770	92.2 (87.2–95.3)	45%, 0.02	89.9 (79.2–95.4)	93.7 (86.5–97.1)	0.33	93.8 (4.1–100)	93.1 (83.1–97.4)	0.84	92.9 (85.0–96.7)	90.5 (73.1–97.1)	0.52
Procedure duration (min)	697	80.4 (66.6–94.2)	96%, < 0.01	85.8 (54.0–117.7)	78.0 (65.4–90.7)	0.66	68.5 (66.8–86.1)	65.7 (49.8–81.6)	0.26	81.4 (65.8–97.1)	77.1 (10.5–143.7)	0.90
Recurrence rate (%)	737	2.0 (0.0–5.1)	0%, 0.94	3.7 (1.6- 8.3)	0.5 (0.0–7.6)	0.13	2.2 (0.0–90.4)	3.9 (0.6–21.4)	0.70	1.6 (0.4–6.6)	2.2 (0.1–32.3)	0.77
Total adverse events rate (%)	708	11.5 (8.7–14.9)	0%, 0.94	15.1 (11.1–20.1)	10.4 (7.3–14.7)	0.06	12.1 (0.4–82.0)	11.0 (6.5–18.1)	0.76	13.2 (10.1–17.0)	8.9 (0.0–97.4)	0.37
Length of Hospitalization (days)	206	4.2 (2.0–6.4)	98%, < 0.01	2.2 (1.0–3.4)	5.6 (2.4–8.8)	0.05	–	4.2 (1.1–7.3)	–	5.0 (1.2–8.8)	2.9 (2.3- 3.4)	0.27
*Statistically significant *P* < 0.05.												

### Second recurrence

**Fig. 3:**
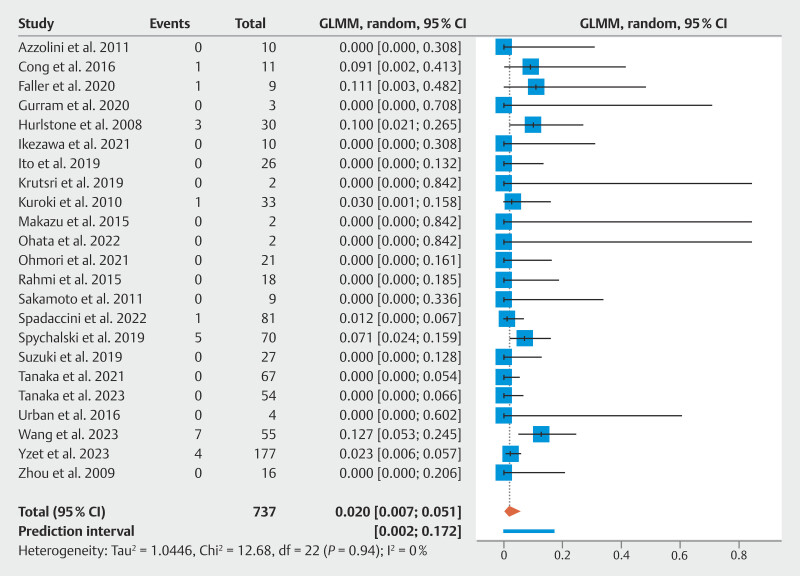
Forest plot of the proportion of local or distant recurrences in patients undergoing endoscopic submucosal dissection for residual or recurrent colorectal neoplastic lesions.

Second recurrence rate was reported in 23 studies, representing 770 lesions (forest plot provided in Fig. 3 ). The pooled estimate of second recurrence rate was 2.0% (95% CI 0.7–5.1%) with low statistical heterogeneity ( *I* ^2^ = 0%). There was no statistically significant difference ( *P* = 0.13) in second recurrence rates between procedures performed in Asian (0.5%, 95% CI 0.0–7.6%, *I* ^2^ = 0%) vs non-Asian countries (3.7%, 95% CI 1.6–8.3%, *I* ^2^ = 7%). Neither the experience of the endoscopist nor the size of the recurrent lesion was associated with differences in second recurrence rates for neoplasia ( Table 2 ).

### Adverse events

**Fig. 4:**
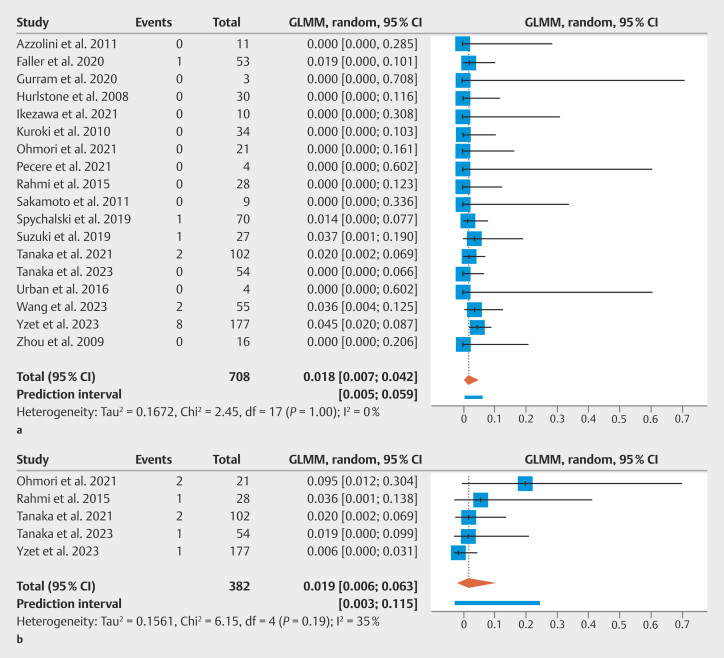
Forest plot of the proportion of adverse events in patients undergoing endoscopic submucosal dissection for residual or recurrent colorectal neoplastic lesions. **a** Delayed bleeding. **b** Delayed perforation.

Combined AEs occurred in 12.4% of ESDs (9.6%-16.0%) for residual or recurrent lesions, with low heterogeneity ( *I* ^2^ = 0%). Combined AEs did not occur more frequently in non-Asian countries (15.1%, 95% CI, 11.1–20.1%, *I* ^2^ = 0%) compared with those performed in Asian countries (10.4%, 95% CI 7.3–14.7%, *I* ^2^ = 0%) in this study ( *P* = 0.06). Likelihood of any AE occurring did not differ based on endoscopist experience or lesion size. The most common AE was intra-procedure perforation (6.2%, 95% CI 3.7–10.1%, *I* ^2^ = 0%). Pooled estimates of delayed perforation and bleeding were 1.9% (0.6–6.3%) and 1.8% (0.7–4.2%), respectively ( Fig. 4 ). The rate of salvage surgery following ESD was 1.3% (0.6–3.0%). Pooled incidences of all individual AEs are provided in **Supplementary Table 4** .

### Procedure duration and length of hospitalization

Estimated procedure duration was 80.4 minutes (95% CI 66.6–94.2 minutes, *I* ^2^ = 96%) and did not differ significantly based on study location, lesion size, or endoscopist experience. Pooled hospitalization length following ESD was 4.2 days (95% CI 2.0–6.4 days, *I* ^2^ = 98%) ( Table 2 ).

### Risk of bias assessment

Detailed risk of bias assessments are provided in Table 3 . Most studies (17/25) were deemed to be at serious risk of bias, primarily due to retrospective designs not permitting detailed adjustment for selection bias. Three studies were found to be at critical risk. There was visual and statistical evidence of publication bias for the combined AE outcome as well as intraoperative bleeding and perforation, with results of Egger’s tests and corresponding funnel plots provided in the **Supplementary Table 5** and **Supplementary Fig. 1** , **Supplementary Fig. 2** , and **Supplementary Fig. 3** .

**Table:** **Table 3** Quality assessment of included studies using ROBINS-I tool for observational studies.

**Study**	**Confounding**	**Selection of participants**	**Classification of intervention**	**Deviations from intended intervention**	**Missing data**	**Measurement of outcome**	**Reported results**	**ROBINS-I score**
Azzolini 2011	3	1	1	1	1	1	4	4
Cong 2016	3	3	1	1	1	1	0	3
Faller 2020	1	1	1	1	1	1	1	1
Gurram 2020	1	1	1	1	1	1	1	1
Hurlstone 2008	1	1	1	1	1	1	3	3
Ikezawa 2021	1	1	1	1	1	1	1	1
Ito 2019	1	3	1	1	2	1	1	3
Krutsri 2019	1	3	1	1	1	1	1	3
Kuroki 2010	1	3	1	1	2	1	1	3
Makazu 2015	3	3	1	1	1	1	1	3
Ohata 2022	1	1	1	1	1	1	1	1
Ohmori 2021	1	3	1	1	1	1	1	3
Pecere 2021	1	3	1	1	1	1	1	3
Rahmi 2015	1	3	1	1	1	1	1	3
Sakamoto 2011	1	3	1	1	1	1	4	4
Spadaccini 2022	1	3	3	1	1	1	1	3
Spychalski 2019	1	3	1	1	1	1	1	3
Suzuki 2019	1	3	1	1	1	1	1	3
Tanaka 2021	1	3	1	1	2	1	1	3
Tanaka 2023	1	1	1	1	1	1	1	1
Urban 2016	1	1	1	1	1	1	4	4
Wang 2023	1	3	1	1	1	1	1	3
Yang 2019	1	3	1	1	2	1	1	3
Yzet 2023	1	3	3	1	1	1	1	3
Zhou 2009	1	3	1	1	1	1	1	3
Risk of bias assessment: 0, no information; 1 low; 2 moderate; 3 serious; 4 critical.								

## Discussion

In this systematic review and meta-analysis, we reported the effectiveness, safety, and resource implications of ESD performed for residual or recurrent colorectal lesions. There are several key findings that collectively suggest that this approach is both effective and safe overall, which could lend to its feasibility and acceptability on a broad scale, although this needs to be considered in the context of overwhelmingly retrospective evidence to date.

Although ESD has been a mainstay of endoscopic resection in Asia for several years, the technique is relatively newer in the Western world. The main driver for development of ESD was the need for an endoscopic approach to be able to afford en bloc resection, and, therefore, R0 resection – an outcome that is impossible for EMR to achieve for most lesions > 15 to 20 mm. High-quality studies have confirmed high rates of en bloc resection for ESD of large colorectal lesions > 90% to 95% 21 38 , which have in turn corresponded to low recurrence rates with this approach at < 1% at expert centers 21 . The key to achieving low recurrence rates was reinforced to be en bloc resection in these studies, with piecemeal resection, even with ESD, being significantly correlated with recurrence, with an associated hazard ratio of over 8 21 . With increasing availability and expertise of ESD in the West, some have suggested that ESD could even be considered a viable alternative to EMR in primary resection of large colorectal adenomas without features suggestive of submucosal invasion based on these improved recurrence rates and the possibility of fewer follow-up endoscopic surveillance exams compared with EMR 39 40 .

Although ESD has gained favor in primary resection of colorectal lesions, relatively little is known about its role in resection of residual or recurrent lesions of the colorectum – lesions previously resected by either endoscopic or surgical approaches. In such lesions, submucosal fibrosis from previous resection often increases the technical difficulty of repeat endoscopic resection 41 . Reasons for this include a relative inability to expand the submucosal space with fluid and/or properly visualize submucosal planes 42 . Encouragingly, pooled effectiveness and safety data from our meta-analysis demonstrate that performance of ESD for residual lesions is actually comparable to its performance for primary colorectal lesions. Specifically, from a meta-analysis of outcomes following ESD as the initial treatment modality for colorectal lesions 43 , R0 resection occurred at a rate of 82.9% (95% CI 80.4%-85.1%), compared with our pooled value of 80.7% (95% CI 72.7%-86.7%), and en bloc resection was achieved in 91.0% of cases (95% CI 89.2%-92.5%) versus in 92.2% of residual/recurrent lesions in our study (95% CI 87.2–95.3%). The need for salvage surgery, having been reported as 1.0% in primary ESD applications (95% CI 0.4%-2.3%) was similar to our reported rate of 1.3% (95% CI 0.6%-3.0%). In terms of AEs, rates of delayed bleeding were 2.7% (95% CI 2.2%-3.2%) for primary lesions versus 1.8% (95% CI 0.7%-4.2%) in our study, and rates of perforation were 5.2% (95% CI 4.4–6.1%) and (6.2%, 95% CI 3.7%-10.1%, *I* ^2^ = 0%), respectively. Finally, the rate of second recurrence was 2.0% (95% CI 1.3%-3.0%) for primary resections versus 2.0% (95% CI 0.7%-5.1%) for residual or recurrent lesions 43 . Although it is prudent to avoid placing too much emphasis on these comparisons, given important underlying differences in patients being studied and the robustness of the study designs, it is nevertheless encouraging to see that outcomes of ESD for residual or recurrent colorectal lesions are well within the range of what would be deemed clinically acceptable even for primary lesions.

The question then arises regarding what approach is optimal for recurrent or residual colorectal lesions. In a recent randomized trial, ESD was compared with EMR for primary resection of lesions ≥ 25 mm in the colon 39 . Absolute recurrence rates were low for both techniques, resulting in only nine recurrences of 318 resected lesions, and of these recurrences, most were treated with either cold snare polypectomy or hot polypectomy, with only one requiring a hybrid technique that included ESD 39 . In an observational study of 74 patients with residual or recurrent colorectal lesions following initial endoscopic resection, ESD was demonstrated to be the favorable approach for patients with cancerous recurrence in terms of en bloc resection and second recurrence, although these differences were deemed to be less clinically significant for patients with recurrence of adenoma only 17 .

Conversely, in a separate comparative observational study, although ESD and underwater EMR both achieved low second recurrence rates for residual or recurrent lesions in the colon, EMR was associated with shorter procedure time, decreased hospitalization length, and decreased risk of delayed perforation 44 . Therefore, several factors, including larger size of the recurrent lesion, presence of obvious scarring or fibrosis, and/or advanced pathology, could lead endoscopists to select ESD for residual or recurrent lesions, whereas EMR, hot polypectomy, or even cold snare polypectomy can be reasonably selected as an effective and safe option for low-grade diminutive recurrences in favorable positions with minimal or no fibrosis.

Our meta-analysis has many strengths. Our comprehensive electronic and manual search strategies enabled inclusion of relevant studies and decreased the likelihood of missing primary studies. We also attempted to create as homogeneous a cohort as possible by employing rigid eligibility criteria to guide study inclusion, and the generally low measures of statistical heterogeneity we observed support this. As an example, we excluded studies reporting on “metachronous” lesions, given the lack of clarity associated with this term. Finally, despite the clinical relevance and importance of our study question, to our knowledge, ours is the first study to systematically review and meta-analyze available data on outcomes of ESD for recurrent or residual colorectal neoplastic lesions.

In addition to these strengths, we acknowledge that our study also has important limitations. First, despite the low statistical heterogeneity observed for most analyses, there was considerable heterogeneity observed in the analysis of pooled incidence for our primary outcome of R0 resection. None of the subgroup analyses we proposed to explore potential sources of this heterogeneity yielded any statistically significant results, and therefore, there are still unexplained sources of heterogeneity. Other valuable subgroup analyses, including by pathology or morphology, could not be conducted due to lack of available patient-level data in the preponderance of original studies. Second, despite including all relevant available data, there were still a relatively low total number of patients included in our study (800 for our primary outcome), meaning that it is difficult to draw sweeping conclusions based on these data. Despite this, the CIs we observed for our pooled incidences of relevant outcomes were narrow enough to draw high-level conclusions regarding the effectiveness and safety, and therefore the feasibility and acceptability, of ESD within this clinical context. Third, several of the input studies we included were at high risk of bias due to failure to adjust for potential confounders (e.g. comorbidities, tumor size, and pathological differentiation). Fourth, there was a paucity of primary studies directly comparing ESD with other approaches such as EMR or surgery for recurrent or residual lesions, as discussed above, which precluded performance of pairwise comparisons, and therefore, limits the widespread generalizability of our findings or their ability to inform clinical guidance. Fifth, the observational design of all included studies means that selection bias also needs to be considered. For instance, patients with recurrent lesions with worrisome associated features could have been referred for surgery at greater rates rather than undergoing ESD and being included in our input studies, and these patients could have been at higher risk of experiencing poorer curative outcomes and/or AEs. This factor must be emphasized and should again limit the scope of conclusions one can draw based on these results. Finally, most studies we included were carried out at single, high-volume centers with longstanding ESD expertise, and most studies were performed in Asia, which collectively also limits the generalizability of our findings. For instance, length of hospitalization is likely to vary considerably between Asian and Western practices. More research is needed both in Western settings and in patients undergoing ESD performed by a wide range of providers (in terms of their training backgrounds, cumulative experience levels, career stages, practice settings, and procedural volumes, among other factors).

## Conclusions

In summary, the available evidence we synthesized suggests that ESD is both an effective and safe treatment modality for recurrent or residual colorectal neoplastic lesions, with a procedure profile that appears comparable to ESD for primary resection in the same clinical setting. Although available data are currently limited to observational studies which could have significant selection bias, especially given that many studies were performed in Asia at high-volume ESD centers, our results suggest overall effectiveness and safety of ESD in this setting. More comparative and ideally prospective studies are needed to assess performance of ESD in this setting compared with EMR or surgery as alternatives and to determine the acceptability and feasibility of performing ESD for this indication in a widespread manner.

## Supplementary Material

Supplementary Material Zusatzmaterial
